# The Association of Inflammatory Biomarker of Neutrophil-to-Lymphocyte Ratio with Spontaneous Preterm Delivery: A Systematic Review and Meta-analysis

**DOI:** 10.1155/2021/6668381

**Published:** 2021-02-01

**Authors:** Sina Vakili, Parham Torabinavid, Reza Tabrizi, Alireza Shojazadeh, Nasrin Asadi, Kamran Hessami

**Affiliations:** ^1^Infertility Research Center, Shiraz University of Medical Sciences, Shiraz, Iran; ^2^Pediatric Urology and Regenerative Medicine Research Center, Section of Tissue Engineering and Stem Cells Therapy, Children's Hospital Medical Center, Tehran University of Medical Sciences, Tehran, Iran; ^3^Non-Communicable Diseases Research Center, Fasa University of Medical Sciences, Fasa, Iran; ^4^Clinical Research Development Unit of Vali Asr Hospital, Fasa University of Medical Sciences, Fasa, Iran; ^5^Health Policy Research Center, Institute of Health, Shiraz University of Medical Sciences, Shiraz, Iran; ^6^Student Research Committee, Shiraz University of Medical Sciences, Shiraz, Iran; ^7^Maternal-Fetal Medicine Research Center, Shiraz University of Medical Sciences, Shiraz, Iran

## Abstract

**Background:**

Neutrophil-to-lymphocyte ratio (NLR), as an inflammatory biomarker, has been investigated in several studies for early prediction of preterm delivery. However, their findings seem to be controversial. Thus, this systematic review and meta-analysis was conducted to evaluate the role of NLR in predicting preterm delivery as compared to term controls.

**Methods:**

PubMed, Web of Science, Embase, Scopus, and Google Scholar were systematically searched from inception up to December 2020. Interstudy heterogeneity was assessed using Cochrane's *Q* test and the *I*^2^ statistic. The random-effects model was employed to pool the weighted mean differences (WMDs) and the corresponding 95% confidence intervals (CIs).

**Results:**

Out of a total of 4369 recodes, fifteen articles including 3327 participants were enrolled. The meta-analysis finding using the random-effects model produced a pooled estimate suggesting a significantly higher NLR (WMD = 1.23, 95% CI: 0.40–2.07) in women with preterm delivery (*P* = 0.01). We found significant heterogeneity across the included studies (*P* < 0.001, *I*^2^ = 92.33%). However, interstudy heterogeneity exists mainly due to differences in the definition of preterm delivery (*I*^2^ = 0.0%). In the metaregression analysis, there was no significant effect of publication year (*B* = −0.288, *P* = 0.088), total sample size (*B* = −0.002, *P* = 0.276), and the mean age of cases (*B* = −0.06, *P* = 0.692) on the association between NLR and preterm delivery.

**Conclusion:**

The results of this meta-analysis revealed that the NLR value is higher in patients with preterm delivery. The NLR could be a useful biomarker for predicting preterm delivery; however, further prospective case-control studies are required to produce stronger evidence.

## 1. Introduction

Preterm delivery, defined as delivery before 37 weeks of gestation, is a major challenge in obstetrics and children's healthcare. It has been estimated that 5 to 18% of all pregnancies end up in preterm delivery which poses an extensive healthcare burden mainly due to neonatal morbidity and mortality [1, 2]. Accordingly, about 15 million neonates are born preterm each year in the world out of whom one million die [2]. In addition to the increased risk of mortality, it has been shown that children born preterm suffer from various short- and long-term morbidities and adverse outcomes such as neurological deficits, learning disabilities, and respiratory problems [3]. In the United States, the economic burden associated with preterm birth is estimated to be more than 26 billion dollars annually with an average of 51,600 dollars per each preterm newborn [4].

The preterm delivery could be medically induced due to maternal or fetal indications; however, most of them (approximately 70%) are spontaneous with no apparent cause [5]. Human childbirth and delivery have a well-known association with inflammatory processes [6]. Since inflammation is suggested to be heavily involved in initiating labor in both preterm and term deliveries [7, 8], previous reports have focused on the alteration in leukocyte counts in order to figure out the correlation between different types of leukocytes and risk of preterm delivery [8, 9]. The number of macrophages has been shown to increase in response to both term and preterm deliveries, but neutrophils are mainly abundant in the decidua of patients with preterm delivery [10]. Thus, it could be hypothesized that markers related to neutrophil may act as a predictive measure for those at risk of preterm delivery.

In recent years, the neutrophil-to-lymphocyte ratio (NLR) has emerged as a novel potential inflammatory biomarker and proved to be correlated with various adverse outcomes in different diseases, especially in obstetric complications such as preeclampsia [11], gestational diabetes mellitus [12], and intrahepatic cholestasis of pregnancy [13]. The NLR can be obtained from a simple complete blood count test which is an inexpensive, well-accessible, and easily performed laboratory test available in many clinical settings. Regarding the predicting value of complete blood count, a recent study showed that maternal serum red blood cell distribution, NLR, white blood cell count, absolute lymphocyte cell count, and the absolute neutrophil cell count profile could guide clinicians in predicting the time of birth in threatened preterm labor [14]. Although there are a number of reports assessing the NLR value for the pregnancies complicated with preterm delivery, the findings seem to be conflicting [15–17].

Since these controversial findings may be a result of variations in the number of participants, study design, etc., a meta-analysis seems necessary to provide a more reliable conclusion on this challenging issue. Accordingly, we conducted this systematic review and meta-analysis, for the first time, to evaluate the relationship between NLR and spontaneous preterm delivery.

## 2. Materials and Methods

We conducted and reported our study according to the Preferred Reporting Items for Systematic Reviews and Meta-Analyses (PRISMA) checklist.

### 2.1. Search Strategy

Literature searches were conducted systematically from databases including PubMed, Web of Science, Embase, and Scopus and Google Scholar search engine from inception up to April 2020, and then, the search was updated again in December 2020. We identified studies examining the association between NLR and preterm delivery using both MeSH terms and any relevant keywords from databases: (“neutrophil to lymphocyte ratio” OR “neutrophil-to-lymphocyte ratio” OR “neutrophil-to-lymphocyte-ratio” OR “neutrophil lymphocyte ratio” OR “neutrophil-lymphocyte ratio” OR “neutrophil-lymphocyte-ratio” OR “lymphocyte” OR “neutrophil” OR “NLR”) AND (“preterm birth” OR “preterm labor” OR “preterm labour” OR “preterm delivery” OR “pre-term” OR “preterm” OR “early birth” OR “early delivery” OR “early labor” OR “early labour”). Our searches were performed in English without date limitation. The reference lists of included studies and previous reviews were manually checked to catch studies not captured by researchers in electronic literature searches. The search strategy is attached as Supplementary file 1.

### 2.2. Study Selection

Two individual researchers (SV and KH) reviewed the titles and abstracts of the identified records after which the full publication papers of the relevant records were retrieved to assess the eligibility. Any discrepancy was resolved through consensus among researchers or discussion with a third author (RT).

Studies were included in our meta-analysis if they met the following inclusion criteria: an original human research with an observational design (cross-sectional, case-control, or cohort), examining the association between NLR and spontaneous preterm delivery, the mean and standard deviations (SDs) of NLR being reported, or being able to calculate and extract the values in both case and control groups. Studies that were not available as a full article or did not have a control group were excluded. Furthermore, studies reporting data on induced preterm deliveries were also excluded.

### 2.3. Data Extraction and Quality Assessment

The information extraction was performed by a researcher (KH), and again, another researcher (RT) independently checked the extracted data for accuracy. The following information from included studies was extracted; author's name, study location, publication year, study method, sample size (in case/control groups), the participants' characteristic, the mean maternal age (in case/control groups), and the key outcome data on mean (SD) of NLR in both case and control groups. The data abstraction sheets in Excel software were used for data extraction.

### 2.4. Quality Assessment

The quality assessment of included observational studies was performed using the Newcastle-Ottawa Scale (NOS). This scale deals with three aspects of selection, comparability, and exposure/outcome. A NOS score of 5 or more for cross-sectional designs and a NOS score of ≥7 for case-control or cohort designs were considered a good quality study.

### 2.5. Statistical Analysis

All statistical analyses in our study were conducted using the STATA 16.0 software (STATA Corp, College Station, Texas). Weighted mean difference (WMD) was considered a summary effect size (ES). Heterogeneity across the included studies was quantified using the chi-squared test and *I*^2^ statistic. *P* ≤ 0.1 with *I*^2^ value ≥ 50% represented substantial heterogeneity. Based on the existence of heterogeneity among studies, we applied a random-effects model to combine WMDs. Due to the small number of the included studies, finally, we used the Hartung-Knapp adjustment. Subgroup and sensitivity analyses were conducted to examine the source of heterogeneity across the included studies. We also used metaregression analyses to determine whether the year of publication, mean age of cases, and total sample size in each study were associated with the estimated WMD and, therefore, were a potential source of heterogeneity across the included studies. Egger's and Begg's tests were used quantitatively to detect any potential publication bias in our meta-analysis.

## 3. Results

### 3.1. Search Strategy and Characteristics of Included Studies

**Figure 1:**
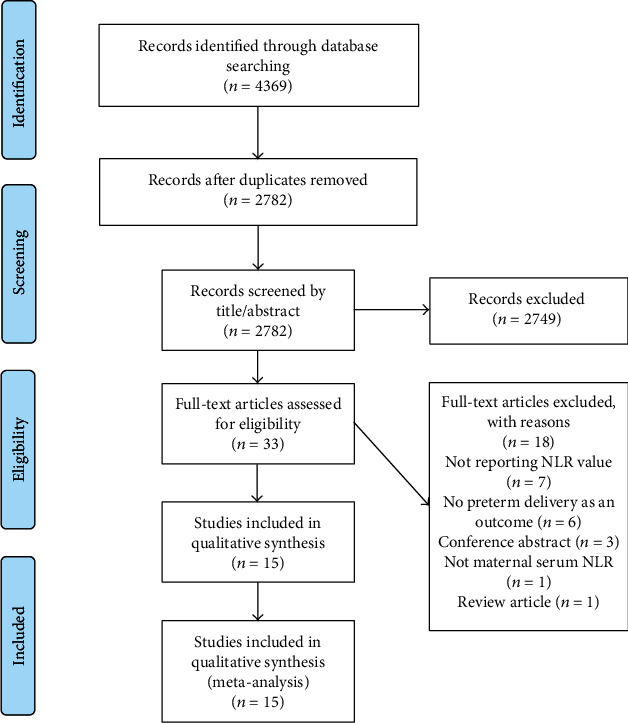
The flowchart of literature search and study identification.

After removing duplicate and irrelevant citations, the full-text paper of 33 out of 4369 records was retrieved for further assessment based on inclusion criteria. Of these, 18 did not appropriately address the desired outcome, so they were excluded. This meta-analysis was conducted finally based on 15 eligible studies [15–29].  Figure 1 displays the flowchart of literature search and study selection.

Eleven of included articles used case-control design [15–19, 21, 22, 25, 27–29], three used cross-sectional design [20, 24, 26], and one used cohort design [23]. Together, these articles involved data on 3327 participants of whom 1309 were in the case group and 2018 in the control group.

Seven articles were undertaken in Turkey [16–18, 22, 25, 26, 28], three in South Korea [23, 27, 29], and five from elsewhere [15, 19–21, 24]. Thirteen articles [15, 17–22, 24–28] used the preterm delivery definition as birth before 37 weeks of gestation and two used before 34 weeks of gestation [16, 23]. The included articles were published from 2011 to 2020. The characteristics of included articles are summarized in Table 1.

### 3.2. NLR for Predicting Preterm Delivery

**Figure 2:**
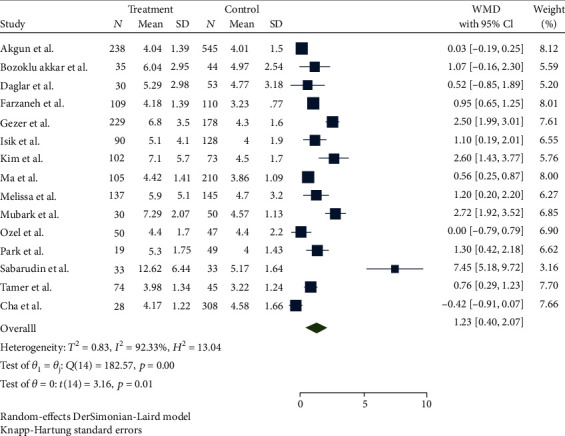
The forest plot of meta-analysis of the association between NLR and preterm delivery.

The forest plot for the association between NLR and preterm delivery is shown in Figure 2. The meta-analysis finding using the random-effects model produced a pooled estimate suggesting a significantly higher NLR (WMD = 1.23, 95% CI: 0.40–2.07) in women with preterm delivery (*P* = 0.01). The corresponding *P* value and *I*^2^ for interstudy heterogeneity tests were significant (*P* < 0.001, *I*^2^ = 92%, 95% CI: 89%-95%).

### 3.3. Source of Heterogeneity

Subgroup analyses were conducted to assess the source of heterogeneity based on the following potential mediator variables: definition of preterm (before 37 weeks vs. before 34 weeks), study design (case-control vs. cross-sectional vs. cohort), and country (Turkey vs. South Korea vs. other countries). As reported in Table 2, the association between NLR and preterm delivery remained significant in subgroups of preterm birth < 37 weeks, studies designed as case-control, cohort, and in Turkey. However, the interstudy heterogeneity mainly is due to the different definitions of preterm delivery (*I*^2^ = 0.0%).

The pre- and postsensitivity analysis mean difference was not statistically significant (pretest WMD = 1.23, 95% CI: 0.40–2.07; posttest WMD = 0.80, 95% CI: 0.24-1.37).

In the metaregression analysis, there was no significant effect of publication year (*B* = −0.28, adjusted *R*^2^ = 0.0%, *P* = 0.088), total sample size (*B* = −0.002, adjusted *R*^2^ = 0.0%, *P* = 0.276), and the mean age of cases (*B* = −0.06, adjusted *R*^2^ = 10.53%, *P* = 0.692) on the association between NLR and preterm delivery.

### 3.4. Publication Bias

**Figure 3:**
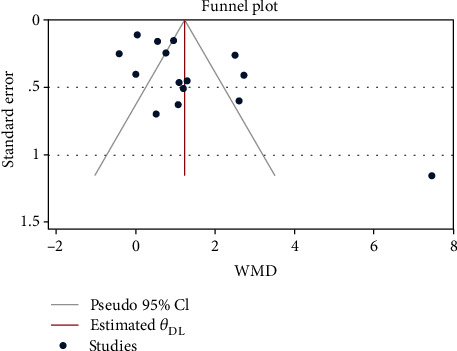
Funnel plot of the association between NLR and preterm delivery.

The funnel plot is shown in Figure 3. This was confirmed when statistically assessed using Begg's and Egger's tests (*P* Begg′s test = 0.235, *P* Egger′s test = 0.013). As there was evidence of publication bias based on our tests, after using the trim and fill method, we found no significant changes in the pooled WMD between before and after including the censored studies.

## 4. Discussion

To the best of our knowledge, this systematic review and meta-analysis is the first to collate evidence on the correlation between NLR value and risk of preterm delivery. Regarding the main findings of our study, NLR values were significantly higher in the pregnancies with preterm delivery as compared with healthy full-term controls. Further, the findings of this meta-analysis did not change after subgroup analyses based on study design and definition of preterm delivery (34 and 37 weeks).

The relationship between inflammation and preterm delivery has been widely studied in terms of factors such as proinflammatory cytokines and different subtypes of leukocytes. Various blood and/or amniotic fluid sample parameters have been investigated because of their potential utility in predicting preterm delivery, including high levels of C-reactive protein (CRP), cytokines, and ferritin [30–32].

As noted earlier, inflammation plays a crucial role in triggering preterm delivery, and the best tool for identifying the potential source of the inflammation/infection is amniocentesis. Nevertheless, amniocentesis has been shown to be associated with a 0.5% risk of fetal loss [33], highlighting the need for less invasive predictive methods. The optimal predictive test should ideally be easily accessible, reproducible, and easy to perform throughout pregnancy to provide enough time for further preventative interventions [34]. The advantage of NLR, a proinflammatory biomarker, is that this value can be obtained from a simple complete blood count test with no need to perform any additional laboratory testing [35].

Recent studies have focused on NLR utility in predicting preterm delivery and revealed interesting results. Kim et al. [27] demonstrated that a combined model consisting of cervical length and NLR has a higher diagnostic and predictive value for spontaneous preterm delivery as compared to cervical length alone or other systemic inflammatory markers such as CRP and leukocytes. Another study by Akgun et al. [22] showed that elevated NLR values are associated with preterm deliveries and lower birth weight newborns. The authors of the latter study hypothesized that the maternal hyperinflammatory state, as indicated by elevated NLR, could result in disturbed fetal growth leading to low birth weight and early initiation of labor.

The NLR has been used for predicting and diagnosing a number of perinatal complications. A previous meta-analysis of 3982 patients suggests that the NLR value significantly increases in preeclamptic pregnancies especially in those with severe features, and it could be a useful biomarker for early diagnosis of preeclampsia and investigating its severity [11]. Furthermore, a previous report comparing predictive accuracy of NLR and CRP for suspected late-onset sepsis in preterm neonates showed that NLR is superior to CRP in detecting culture-proven cases of sepsis [36]. Although there is a growing body of evidence highlighting the potential role of various inflammatory markers such as NLR, findings continue to be inconsistent across studies. Nevertheless, the findings of this review article suggest that NLR may be a useful tool for identifying women at risk for preterm delivery.

### 4.1. Implications for Future Research

Various maternal and fetal factors are suspected in the pathogenesis of preterm delivery, which has not been considered by most studies included in this meta-analysis. Furthermore, these data are obtained from mostly retrospective observational studies. Thus, studies with larger sample sizes as well as prospective and randomized designs (if applicable) are necessary for an in-depth assessment of the actual role of NLR for predicting preterm delivery while adjusting for confounding factors.

### 4.2. Strengths and Limitations

To the best of our knowledge, the present systematic review and meta-analysis summarizes, for the first time, the current literature regarding the role of NLR for early identification of women at risk of preterm delivery. This meta-analysis was limited by a relatively small number of nonrandomized studies, significant heterogeneity among them, and not being registered in a systematic review registry. The gestational age for defining the preterm delivery was different across some studies (37 vs. 34 weeks); the gestational age at blood sampling was different also leading to heterogeneity in their findings. Thus, the results should be interpreted with caution. Nevertheless, heterogeneity was explored by conducting subgroup, metaregression, and sensitivity analyses, minimizing the effect of potential confounders.

## 5. Conclusion

This systematic review and meta-analysis confirmed that the NLR can be potentially useful for the prediction of spontaneous preterm delivery. However, there seems to be a great need for larger prospective cohort studies, with more uniformity in the study design with a rigorous methodological assessment to reach a more reliable conclusion.

## Figures and Tables

**Table 1:** Characteristics of included studies assessing the association between NLR and preterm delivery.

Authors	Publication year	Country	Sample size (case/control)	Study type	Defined preterm delivery	Mean age of cases	Quality assessment
Akgun et al. [22]	2017	Turkey	238/545	Prospective case-control	37 weeks	30.67 ± 4.53	High
Akkar et al. [25]	2016	Turkey	35/44	Prospective case-control	37 weeks	25.5 ± 5.6	Low
Cha et al. [29]	2020	South Korea	28/308	Prospective case-control	37 weeks	32.1 ± 4.2	High
Daglar et al. [17]	2016	Turkey	30/53	Prospective case-control	37 weeks	26.4 ± 8.2	Low
Farzaneh et al. [24]	2019	Iran	109/110	Cross-sectional	37 weeks	27.61 ± 5.14	Low
Gezer et al. [28]	2018	Turkey	229/178	Retrospective case-control	37 weeks	25.9 ± 5.1	High
Isik et al. [16]	2015	Turkey	90/128	Retrospective case-control	34 weeks	28.7 ± 5.1	High
Kim et al. [27]	2011	South Korea	102/73	Retrospective case-control	37 weeks	31 ± 3.9	High
Ma et al. [15]	2020	China	105/210	Retrospective case-control	37 weeks	29.93 ± 4.04	High
Melissa et al. [19]	2018	USA	137/145	Retrospective case-control	37 weeks	28.1 ± 5.6	Low
Mubark et al. [21]	2015	Iraq	30/50	Prospective case-control	37 weeks	28.03 ± 4.58	Low
Ozel et al. [26]	2020	Turkey	50/47	Cross-sectional	37 weeks	27.8 ± 4.2	High
Park et al. [23]	2019	South Korea	19/49	Retrospective cohort	34 weeks	34 [25-39]^∗∗^	High
Sabarudin et al. [20]	2016	Indonesia	33/33	Cross-sectional	37 weeks	28.39 ± 5.6	Low
Tamer et al. [18]	2017	Turkey	74/45	Case-control^∗^	37 weeks	22.5 ± 2.1	Low

^∗^Indirectly conceived from methods, ^∗∗^median (range).

**Table 2:** Outcomes of subgroup analysis for association between NLR and preterm delivery.

Subgroups		Number of studies	WMD (95% CI)	*I* ^2^
Definition of preterm delivery	Before 37 weeks	13	1.02 (0.40, 1.63)	91.82%
	Before 34 weeks	2	1.20 (-0.07, 2.47)	0.0%
Study design	Case-control	11	1.11 (0.39, 1.83)	92.72%
	Cross-sectional	3	2.30 (-7.08, 11.68)	94.62%
	Cohort	1	1.30 (0.42, 2.18)	—
Study country	Turkey	7	0.86 (0.04, 1.68)	92.69%
	South Korea	3	1.10 (-2.67, 4.87)	92.87%
	Other	5	1.95 (-0.85, 4.75)	92.93%

## Supplementary Materials

Supplementary Materials Supplementary file 1: sample search strategy for PubMed.
