# Supplementary material for: The Association of Inflammatory Biomarker of Neutrophil-to-Lymphocyte Ratio with Spontaneous Preterm Delivery: A Systematic Review and Meta-analysis
Source: Mediators Inflamm. 2021 Feb 1;2021:6668381. doi: 10.1155/2021/6668381 (PMC7870293; doi:10.1155/2021/6668381)
Supplement: Supplementary Materials — Supplementary file 1: sample search strategy for PubMed. [file 6668381.f1.docx]

Supplementary file 1: Sample search strategy for PUBMED

1 "neutrophil to lymphocyte ratio"

2 "neutrophil-to-lymphocyte ratio"

3 "neutrophil-to-lymphocyte-ratio"

4 "neutrophil lymphocyte ratio"

5 "neutrophil-lymphocyte ratio"

6 "neutrophil-lymphocyte-ratio"

7 "lymphocyte"

8 "neutrophil"

9 "NLR”

10 1 OR 2 OR 3 OR 4 OR 5 OR 6 OR 7 OR 8 OR 9

11 "preterm birth"

12 "preterm labor"

13 "preterm labour"

14 "preterm delivery"

15 "pre-term"

16 "preterm"

17 "early birth"

18 "early delivery"

19 "early labor"

20 "early labour"

21 11 OR 12 OR 13 OR 14 OR 15 OR 16 OR 17 OR 18 OR 19 OR 20

22 10 AND 21
